# Effects of Dietary Ginger (*Zingiber officinale*) Rhizome Powder Supplementation on Productive Performance, Egg Quality, Antioxidant Capacity, and Hepato-Intestinal Morphology in Pre-Peak Xiaoshan Laying Hens

**DOI:** 10.3390/ani15152315

**Published:** 2025-08-07

**Authors:** Debela Bayu Derese, Hanxue Sun, Xihuai Xiong, Ziqing Li, Rahmani Mohammad Malyar, Lizhi Lu, Fangxiong Shi

**Affiliations:** 1 College of Animal Science and Technology, Nanjing Agricultural University, Nanjing 210095, China; debela.bayu1@ambou.edu.et (D.B.D.); sunhx@zaas.ac.cn (H.S.); xiaoxiongxxh@outlook.com (X.X.); zqli98@163.com (Z.L.); 2021205039@stu.njau.edu.cn (R.M.M.); 2 Institute of Animal Husbandry and Veterinary Medicine, Zhejiang Academy of Agricultural Science, Hangzhou 310021, China; lulz@zaas.ac.cn; 3 Department of Animal Science, School of Agriculture, Ambo University, Ambo P.O. Box 19, Ethiopia; 4 Faculty of Veterinary Science, Nangarhar University, Jalalabad 2601, Afghanistan

**Keywords:** antioxidant enzymes, egg quality, ginger powder, hepato-intestinal morphology, Xiaoshan hens

## Abstract

Ginger powder (GP) has antioxidant properties and can be a suitable alternative to antibiotics in laying hen diets; however, research on its effects remains limited. Therefore, our study aimed to evaluate the impact of dietary GP supplementation on production performance during the pre-peak production stage. A total of 270 hens, 18 weeks old and averaging 1.83 ± 0.03 kg, were divided into three groups: control (CN, basal diet), CN + 5 g/kg GP (T1), and CN + 10 g/kg GP (T2), with six replicates of 15 hens each, in a 10-week feeding trial. Dietary GP had no significant effect on feed intake (*p* > 0.05), but it dose-dependently improved laying rate, egg mass, and feed conversion ratio (*p* < 0.05). Egg quality parameters, including albumen height, Haugh unit, eggshell thickness, and strength, were also linearly improved with GP supplementation (*p* < 0.05). Dietary GP linearly enhanced the antioxidant status of hens (*p* < 0.01) and reduced malondialdehyde activity (*p* < 0.0001). Furthermore, 10 g/kg GP supplementation slightly improved gizzard index and liver morphology, and it linearly enhanced intestinal morphology (*p* < 0.01). These findings suggest that 10 g/kg GP supplementation can improve the productivity and health of laying hens.

## Simple Summary

This study evaluated the effects of dietary supplementation with ginger powder on production performance, egg quality, antioxidant status, and hepato-intestinal morphology in pre-peak Xiaoshan laying hens. A total of 270 hens received different levels of ginger powder supplementation (0, 5, 10 g/kg) over 10 weeks. The results showed that although dietary ginger powder did not significantly influence feed intake, it improved laying rate, egg mass, feed conversion ratio, and some egg quality parameters. Additionally, dietary ginger powder supplementation enhanced the hens’ antioxidant status and gizzard index and positively affected the morphology of the liver and intestines. These findings suggest that dietary inclusion of 10 g/kg ginger powder can enhance productivity and physiological health in young laying hens, offering a potential natural alternative to in-feed antibiotics.

## 1. Introduction

The productive performance of poultry is a key factor in the sustainability and success of the poultry industry. However, with the ongoing intensification of poultry production, hens are increasingly at risk from heat, immune, and oxidative stress, which can directly or indirectly impair their performance [1,2,3,4]. Additionally, stress caused by feed composition, microbiological issues, and management challenges is the primary cause of oxidative stress [5]. Therefore, it is recommended that the antioxidant defense system in hens be supported through nutritional interventions [6].

Antibiotics have been widely used as feed additives in poultry to improve performance. However, they have been found to increase levels of antibiotic residues in poultry products, promote drug resistance in bacterial populations, and pose various environmental risks [7,8]. Consequently, policy restrictions on antibiotic use have been effective, prompting the search for alternative feed supplements [9]. This has made the poultry industry seek new feed additives that can enhance poultry health and performance while ensuring product safety and quality for consumers [10,11]. In this context, researchers are focusing on medicinal herbs rich in phytochemicals as potential substitutes for antibiotics in poultry diets [2,12,13]. One such herb is ginger, the rhizome of *Zingiber officinale*, a member of the *Zingiberaceae* family native to Southeast Asia. It has a long history of use in traditional and modern Chinese medicine [14,15]. Widely used as a spice and medicinal plant, it is rich in bioactive compounds like gingerol, shogaols, paradols, zingerone, and essential oils, which have antioxidant, anti-inflammatory, anticancer, digestive-enhancing, and antimicrobial properties [16]. Besides its bioactive compounds, ginger is a good source of Fe, Mg, Ca, and vitamin C [14,17]. These properties of ginger and its products suggest that dietary ginger as a supplementary feed to the poultry diet may positively influence the productive efficiency of hens.

Currently, phytogenic feed additives derived from ginger and other herbs are commonly used in poultry diets. They are claimed to improve hen performance by enhancing antioxidant status, antimicrobial activity, immunity, gut health, digestion, and nutrient absorption [18]. Studies have shown that ginger powder supplements (5–15 g/kg) can positively affect laying rate, egg quality, egg weight, and the antioxidant status of laying hens [19,20]. Dietary ginger powder (GP) can also help alleviate oxidative stress and has been reported to reduce the adverse effects of molt on reproductive efficiency [21]. Further research indicates that dietary GP (15 g/kg) enhances antioxidant capacity and productivity in quails [12]. Based on these findings, we hypothesize that the bioactive compounds in dietary GP may boost the productivity of Xiaoshan hens during the pre-peak period through improved antioxidant capacity and liver and gut health, which, in turn, could enhance feed digestion and nutrient absorption [22]. Furthermore, different researchers suggest different optimum ranges of ginger powder supplementation levels in poultry [19,23]. According to [24], increasing ginger powder inclusion (5–15 g/kg) in the diet results in reduced feed intake in poultry. In our pre-experiment test, we also noticed a significant decline in the average daily feed intake of hens fed 15 g/kg GP. In addition, as the diet during the early life of hens significantly affects the development of reproductive organs and subsequently impacts their laying cycle later on [25], it is imperative to manipulate the diet during the pre-peak period [26]. The Xiaoshan hen is an important Chinese indigenous dual-purpose breed originating from Hangzhou, China, renowned for its tasty meat [27]. It is among the top three Chinese indigenous breeds prioritized for conservation [28].

However, despite its significant contribution to the poultry diet, detailed studies on the specific biochemical and histological mechanisms by which dietary GP supplementation influences the productive performance of hens, especially during the pre-peak laying stage, are lacking. Therefore, the present study aimed to investigate the effects of dietary GP supplementation on productive performance, egg quality, antioxidant enzyme status, and the hepato-intestinal morphology in Xiaoshan hens during the pre-peak laying stage.

## 2. Materials and Methods

### 2.1. Experimental Design, Hens, and Management

A total of 270 Xiaoshan laying hens, aged 17 weeks with an average weight of 1.83 ± 0.03 kg, were randomly assigned to three groups, with 90 hens in each group. Each treatment was divided into six replicates of 15 adjacent cages, with one hen per cage. The experiment included a 2-week adaptation period followed by 10 weeks of data collection. The three dietary groups were a control (CN, basal diet), CN + 5 g/kg GP (T1), and CN + 10 g/kg GP (T2). Ginger powder used in this research was sourced from Delicious Treasure Beijing Biotechnology Co., Ltd., Beijing, China. The ginger (*Zingiber officinale*) roots were washed, sliced, and sun-dried to a dry matter content exceeding 90%, then ground into a powder (100-mesh sieve). The ginger powder was mixed with the basal diet (5 and 10 g/kg GP) using an automatic mixer (Zhejiang Mushen Machinery Co., Ltd., Hangzhou, China) on the chicken farm. The dietary GP was added on top of the basal diet as a supplement (5 and 10 g/kg), and its chemical composition is presented in Table 1. The selected doses of ginger powder supplements were based on observations during the pre-experiment and previous studies [24]. The hens were housed in two-level conventional cages measuring 42 × 32 × 40 cm (L × W × H) at the Xiaoshan Chicken Breeding Resource Base (Hangzhou, China), equipped with a feeder and automated two-nipple drinkers. The basal diet was prepared according to the guidelines from the National Research Council (NRC) [29] (Table 1). Throughout the experiment, hens had free access to feed (approximately 20 g per hen daily) and water, which were provided daily at 6:30 a.m. The temperature inside the chicken house was maintained between 18 °C and 25 °C, with a relative humidity between 50% and 60%. The lighting schedule was 16 h of light followed by 8 h of darkness. No chickens died during the study.

### 2.2. Productive Performance Measurements

For each replicate group, feed allotment and feed remaining in the feeder (i.e., feed refusal) were weighed weekly to record the average daily feed intake (ADFI). Eggs were collected three times a day (at 9:00 a.m., 1:30 p.m., and 6:00 p.m.) per replicate treatment, and the average egg weight was measured using an electronic balance (±0.01 g accuracy). Eggs with soft shells, frosted shell surfaces, deformations, and extreme weights (≤20 g or ≥60 g) were considered unqualified. To assess the productive performance of the hens, the laying rate (LR), average egg weight (EW), total egg mass (EM), and feed conversion ratio (FCR) were recorded for each replicate. Each hen was weighed every two weeks to monitor changes in body weight. All recorded productive performance data were expressed as the mean of six replicates per group and reported as average values over 2 weeks. All parameters were calculated per replicate group as follows:

$$ADFI\;\left(g\right)=\frac{Weekly\;feed\;offered\;\left(g\right)-\;Weekly\;feed\;leftover\;\left(g\right)}{7\;days}$$

$$LR\;\%=\frac{Total\;number\;of\;eggs}{Total\;number\;of\;hens}\times 100$$

$$EW\;\left(g\right)=\frac{Total\;weight\;of\;eggs\;\left(g\right)}{Total\;number\;of\;eggs}$$

$$EM\;(g/hen/day)=\frac{Total\;weight\;of\;eggs\;\left(g\right)}{Total\;number\;of\;hens}$$

$$FCR\;(g\;feed/g\;egg\;mass)=\frac{ADFI\;\left(g\right)}{EM\;\left(g\right)}$$

### 2.3. Determination of Egg Quality

At the end of the 10-week experimental period, 36 fresh, representative eggs (12 eggs per group) were randomly collected and labelled according to their replicate groups. All eggs were weighed individually using a sensitive electronic balance (OHAUS, Parsippany, NJ, USA) (±0.01 g accuracy) and assessed for quality parameters within 12 h. Egg height (mm) and width (mm) were measured with a 200 mm electronic caliper (Mitutoyo, S-530, Japan) (±0.01 mm accuracy). Eggshells were weighed with a digital scale (Adam, Milton Keynes, UK) (±0.0001 g accuracy). Eggshell thickness was measured using a digital micrometer (Starrett, Athol, MA, USA) at three locations (tip, equator, and broader end), with the average thickness calculated from these measurements. Eggshell strength was determined using an eggshell force reader (EFR-01, Orka, Ramat HaSharon, Israel). Yolk color was assessed at three locations (tip, equator, and broader end) using a yolk color chart (DSM, Heerlen, The Netherlands), and the average was used for analysis. The following formulas were applied to evaluate the egg quality parameters.

$$Yolk\;ratio\;\%=\frac{Yolk\;weight\;\left(g\right)}{Egg\;weight\;\left(g\right)}\times 100$$

$$Albumen\;ratio=\frac{Albumen\;weight\;\left(g\right)}{Egg\;weight\;\left(g\right)}\times 100$$

$$Eggshell\;\%=\frac{Eggshell\;weight\;\left(g\right)}{Egg\;weigtht\;\left(g\right)}\times 100$$

$$Egg\;shape\;index=\frac{Egg\;width\;\left(mm\right)}{Egg\;length\;\left(mm\right)}\times 100$$

$${HU=log10\;(h-1.7\times w}^{0.37}+7.6)$$

where HU = Haugh unit; h = albumen height (mm); and w = egg weight (g).

### 2.4. Sample Collection and Procedures

At the end of the experiment, one representative hen from each of the six replicates (six hens/treatment) was selected and fasted for 12 h for accurate organ weight measurements. Before slaughter, each hen’s live weight was recorded using an electronic scale (±10 g accuracy). Blood samples were collected from the jugular vein and filtered into sterile procoagulant tubes. These samples were centrifuged at 4500 rpm at 4 °C for 15 min to separate the serum, which was stored at −20 °C for subsequent antioxidant analysis. After blood collection, the hens were sacrificed by severing the jugular vein and carotid artery on one side of the neck, following the animal ethics guidelines [30]. Immediately after slaughter, the heart, liver, and gizzard (emptied of digesta contents) were isolated, weighed using an electronic balance (±0.01 g accuracy), and expressed as a relative percentage of the live body weight [31]. For subsequent histological observations, approximately 2 cm long sections of samples were obtained from the middle segments of the liver, duodenum, jejunum, and ileum and treated with 4% paraformaldehyde.

$$Organ\;index\;(g/kg\;BW)=\frac{Organ\;weight\;\left(g\right)}{Live\;body\;weight\;\left(kg\right)}$$

### 2.5. Determination of Serum Antioxidant Enzyme Activity

Serum concentrations of antioxidant enzymes, including superoxide dismutase (SOD), catalase (CAT), glutathione peroxidase (GSH-Px), total antioxidant capacity (T-AOC), and malondialdehyde (MDA), were measured using an assay kit purchased from Beijing Huaying Institute of Biotechnology, Beijing, China. The procedures followed the manufacturer’s instructions.

### 2.6. Morphological Analyses of the Liver and Intestines

Standard protocols were employed for the morphological analysis of the liver and small intestine (duodenum, jejunum, and ileum). After fixation in 4% paraformaldehyde for 24 h, the tissues were soaked and dehydrated through a graded series of ethanol, cleaned with xylene, embedded in paraffin blocks, and sectioned at 5 µm thickness using a microtome. The sections were floated in a warm water bath (37 °C) and mounted on slides. They were deparaffinized with xylene, rehydrated through graded ethanol dilutions, and stained with hematoxylin and eosin (HE). Six slide replicates were examined for each group, and images were captured using a Nikon ECLIPSE E200 microscope (Nikon Instruments Inc., Shanghai, China) at a magnification of 4×. After calibrating the representative image for each group, six measurements (n = 6) were taken for villus height (VH) and crypt depth (CD) using the ImageJ software version 1.54g (National Institute of Health, USA), and the VH:CD ratio was calculated [32]. VH is defined as the vertical distance from the tip of the villus to the crypt junction. The depth of invagination between adjacent crypts is referred to as CD [33]. VH was measured from the apex of the villus to the upper surface of the lamina propria, while CD was measured from the base to the crypt–villus junction [18].

### 2.7. Statistical Analysis

The data were checked for normality using the Shapiro–Wilk test and analyzed through one-way ANOVA with GraphPad Prism version 9.5 and SPSS version 20 (SPSS Inc., Chicago, IL, USA). Data for all parameters were analyzed using the mean value of each replicate as an experimental unit (n = 6). Egg quality data were analyzed using 12 eggs per group, with the average values of 2 eggs from each replicate serving as the experimental unit (n = 6). Intestinal morphology data were analyzed using six measurement values from each representative slide per group as an experimental unit (n = 6). Tukey’s test was used to test the significant differences between the mean values of the control and GP-treated groups. Results are presented as mean ± SEM, and the statistical significance difference was declared at *p* < 0.05, and 0.05 < *p* < 0.10 was considered as a trend towards significance. The statistical regression model in SPSS 20 was also used to evaluate the linear and quadratic relationships between dietary GP supplement dosages (0, 5, and 10 g/kg) for specific parameters.

## 3. Results

### 3.1. Productive Performances

**Figure 1:**
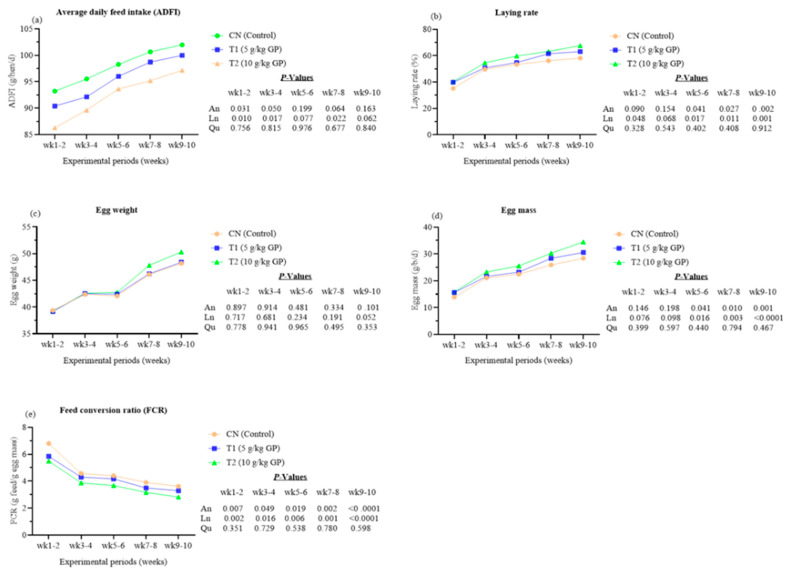
The effects of supplementary GP on the performance of Xiaoshan hens (15 birds per replicate) fed on GP (n = 6). (**a**) Average daily feed intake (g/day/hen). (**b**) Laying rate (%). (**c**) Egg weight (g). (**d**) Egg mass (g/hen/day). (**e**) Feed conversion ratio (g feed/g egg mass). Line graphs show the group means. Abbreviations: ADFI, average daily feed intake; An, ANOVA; d, day; CN, basal diet; FCR, feed conversion ratio; Ln, linear; Qu, quadratic; T1, diet supplemented with 5 g/kg GP; T2, diet supplemented with 10 g/kg GP; wk, weeks of the study period.

The effects of supplementing the diets of Xiaoshan hens with GP on their performance are summarized in Figure 1. During the first to fourth week of the study period, hens in the GP-supplemented groups exhibited a linear decrease in average daily feed intake (ADFI) with increasing dietary GP levels, compared to hens in the CN group (*p* < 0.05). No significant differences were observed between the groups from the fifth to tenth week of the study period (*p* > 0.05). However, during the fifth to tenth week of the study period, hens receiving higher levels of GP supplements showed a linear decreasing trend in ADFI compared to the CN group (*p* < 0.1). During the fifth to tenth week, both laying rate and egg mass in the GP-supplemented groups increased linearly with higher dietary GP levels compared to the CN group (*p* < 0.05). Throughout the study period, supplementing hens’ diets with GP had no significant effect on egg weight compared to the CN group (*p* > 0.05). Nonetheless, during the ninth to tenth week of the study period, a linear increase (*p* < 0.1) in egg weight was observed with higher GP supplementation levels compared to the CN group. Over the entire study, the feed conversion ratio (FCR) of hens supplemented with GP decreased linearly as dietary GP levels increased, compared to the CN group (*p* < 0.05). Notably, during the final four weeks (weeks 7 to 10), FCR decreased linearly with increasing dietary GP levels relative to the CN group (*p* < 0.001).

### 3.2. Egg Quality

The effects of dietary GP supplementation on the egg quality of Xiaoshan hens are presented in Table 2. From the analysis of egg quality, the albumen height, Haugh unit (HU), shell thickness, and shell strength increased linearly in a dose-dependent manner compared to the CN group (*p* < 0.05). However, no significant differences were observed between the groups for the other quality parameters (*p* > 0.05).

### 3.3. Body Weight and Organ Index

The effects of supplementing the diet with dietary GP on the organ indices of Xiaoshan laying hens are summarized in Table 3. The study results showed that the gizzard weight and index increased linearly with rising levels of dietary GP supplementation compared to the CN group (*p* < 0.05). However, body weight changes, heart index, liver weight, and liver index were not significantly affected by different levels of dietary GP supplements compared to the CN group (*p* > 0.05); nonetheless, a linear increasing trend (*p* < 0.1) was observed in heart weight of hens fed increasing levels of dietary GP supplements compared to the CN group.

### 3.4. Liver and Intestinal Morphology

**Figure 2:**
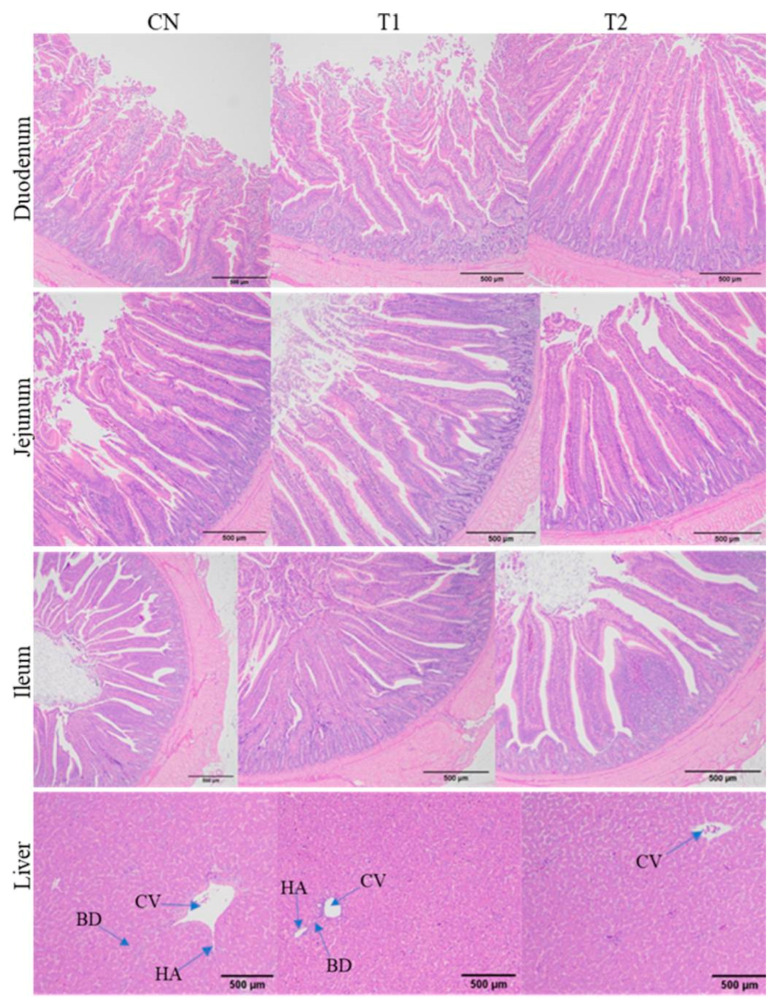
Histological presentation of H & E-stained sections of liver and intestine from laying hens (15 hens per group), (n = 6) fed a basal diet (CN), CN + 5 g/kg GP (T1), and CN + 10 g/kg GP (T2). Scale bar: 500 µm. BD = bile duct, CV = central vein, HA = hepatic artery, S = sinusoids. Liver (CN): normal hepatocytes but dilation of CV with infiltration of lymphocytes around it. Liver (T): normal hepatocytes but infiltration of lymphocytes around CV. Liver (T): normal hepatocytes with narrower CV than the CN group.

The study results (Figure 2) showed that dietary GP supplementation positively affects hens’ liver morphology, although some differences are observed between the groups. The hepatic pathological status indicates that the levels of vacuoles and hepatocytes between the groups are slightly similar and normal, with no observable lipid accumulation in any group. When examining the detailed morphological observations, the liver of hens fed a control diet showed normal hepatocyte morphology with some lymphocyte infiltration around the central vein (CV) and dilation of the CV. Similarly, the liver of hens fed dietary GP (5 g/kg) showed normal hepatocytes, but lymphocyte infiltration around the CV, along with the loss of lining endothelial cells and a narrower CV compared to the CN group, was evident. The liver of hens fed dietary GP (10 g/kg) also showed normal hepatocytes, with a more normal and narrower central vein than that of hens fed the control diet. Regarding intestinal morphology (Table 4 and Figure 2), dietary GP supplementation linearly increased (*p* < 0.01) VH in the duodenum and jejunum, and CD was found to linearly decrease (*p* < 0.05) with increasing levels of GP supplements compared to the CN group. However, in the ileum, VH increased quadratically (*p* < 0.01) and CD decreased quadratically (*p* < 0.05) with higher levels of GP supplements compared to the CN group. Furthermore, dietary GP linearly increased the VH/CD ratio (*p* < 0.01) in all intestinal segments of hens compared to the CN group.

### 3.5. Serum Antioxidant Activity

**Figure 3:**
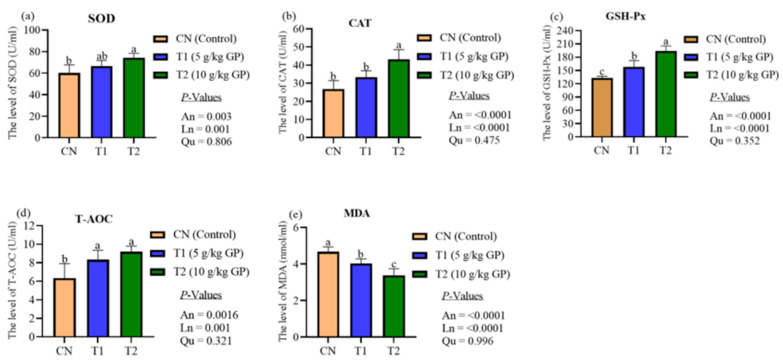
The activity of serum antioxidant enzymes in Xiaoshan hens (15 birds per replicate) fed on GP (n = 6). (**a**) Level of SOD (U/mL). (**b**) CAT (U/mL). (**c**) Level of GSH-Px (U/mL). (**d**) Level of T-AOC (U/mL). (**e**) Level of MDA (nmol/mL). Bar graphs show the group means, with error bars representing the standard error of the mean. Significant differences between groups are indicated by the letters a, b, and c (*p* < 0.05). Abbreviations: An, ANOVA; CAT, catalase; CN, basal diet; GSH, glutathione; Ln, linear; MDA, malondialdehyde; Qu, quadratic; SOD, superoxide dismutase; T1, diet supplemented with 5 g/kg GP; T2, diet supplemented with 10 g/kg GP; T-AOC, total antioxidant capacity.

The effects of dietary GP supplementation on the activity of antioxidant enzymes in laying hens are displayed in Figure 3. The results showed that dietary GP supplementation linearly increased the activity of serum antioxidant enzymes (SOD, CAT, GSH-Px, T-AOC) in hens compared to the CN group (*p* < 0.01). However, the serum concentrations of MDA linearly decreased in hens with increasing levels of dietary GP supplementation compared to the CN group (*p* < 0.0001).

## 4. Discussion

The study aimed to assess the effects of supplementing the diet with GP on production performance, egg quality, antioxidant status, and the morphology of the liver and intestines in hens during the pre-peak stage under normal physiological and non-challenged experimental conditions. The egg production and egg quality of hens are key factors for the profitability and sustainability of the poultry industry. Several researchers have suggested that medicinal herbs could potentially replace antibiotics as dietary supplements for poultry [12,13] because they are rich in active biochemicals and nutrients [14,17]. Therefore, it was hypothesised that GP supplementation improves hen productivity and egg quality by enhancing antioxidant status, liver and intestinal morphology, and feed digestion efficiency.

In the present study, the improvement in hens’ laying rate with increasing levels of dietary GP supplementation (5 and 10 g/kg) indicates that supplementing with 10 g/kg of GP can potentially enhance egg laying during the pre-peak laying stage in a dose- and time-dependent manner. Consistent with these results, several studies have confirmed the positive effects of dietary GP supplementation (10–15 g/kg) on layer performance [2]. Similarly, previous research reported that adding GP (20 g/kg) to the diet positively influenced pre-starter turkey production performance [34], which is a slightly higher dose of dietary GP than that used in our current study. Likewise, egg mass was linearly increased with higher levels of dietary GP supplementation (5 and 10 g/kg), indicating that 10 g/kg dietary GP is most effective during the pre-peak laying stage. Similar to our findings, supplementation with 10 g/kg of dietary GP was claimed to boost egg mass [2]. Furthermore, a slightly higher level of dietary GP (15 g/kg) was reported to increase egg mass without significantly affecting egg weight [5,19], suggesting that the phytogenic premix may help mitigate oxidative stress. However, dietary GP supplementation did not significantly influence the ADFI of hens compared to the CN group, although a decreasing trend was observed with increasing GP levels. The slight reduction in ADFI could be due to ginger’s ability to stimulate digestive enzymes and improve gut morphology, thereby enhancing nutrient absorption, which potentially reduces the hens’ need for higher feed intake to meet metabolic demands [23]. This finding partially contrasts with previous reports where increasing levels of dietary GP (5–20 g/kg) decreased broiler ADFI [35]. The FCR was notably lower in hens fed with dietary GP compared to the CN group, indicating improved feed efficiency. Previous studies suggest that phytogenic feed additives enhance feed efficiency by reducing hepatic fat synthesis and promoting lipid breakdown [36]. The positive effects of dietary GP on feed efficiency may be attributed to bioactive metabolites released during hydrolysis [20]. Overall, the observed improvement in performance among hens fed dietary GP could be linked to significant enhancements in their intestinal morphology, antioxidant status, and gizzard development, as demonstrated in our current study. The discrepancies between this study and others could stem from differences in ginger powder dosage, hen age, breed, or poultry species [37]. The processing temperature levels could influence the amount and effectiveness of ginger’s bioactive compounds [38], which may be different from the current study, where it was slow-dried in the sun before being ground into powder. Additionally, the structure of processed ginger (coarse versus fine powder) may also affect the bioavailability and efficacy of bioactive compounds in hens [39], which may differ from the one we used in the current study, where it was ground into powder (100-mesh sieve). Further research should explore different phases of egg production and varying levels of dietary GP supplementation to provide more comprehensive insights.

Egg quality, indicating a hen’s performance and the nutritional and commercial worth of eggs, impacts the profitability and sustainability of the egg industry [40,41]. Besides genetics, feed type [42], management practices [43], and age, phytogenic feed additives also have been shown to enhance egg quality [44]. In this study, hens fed diets supplemented with GP laid eggs with enhanced albumen height, HU, eggshell thickness, and strength. This result aligns with previous reports suggesting that phytogenic feed additives can enhance egg quality [45,46]. Eggshell thickness is positively correlated with eggshell strength [47,48]. Both are vital indicators of egg quality and influence the eggs’ ability to withstand breakage during transport [49]. These parameters are influenced by the nutrient density of the diet, especially during the early laying phase, with higher nutrient density associated with increased eggshell thickness and strength [50]. Furthermore, the production system has a significant impact on eggshell strength, with higher values reported in eggs from free-range hens [51]. Consistent with our findings, dietary GP supplementation (15 g/kg) [12] and ginger extract supplementation (100 g/t) [20] have been shown to enhance albumen height and HU. Additionally, [21] noted the positive effect of dietary GP supplementation on albumen height in aged hens, suggesting that ginger alleviates oxidative stress and improves egg quality. Albumen height and HU are key indicators of egg freshness [52]. HU is a crucial parameter of egg quality influenced by diet [53], where higher HU values indicate fresher eggs and higher protein content, reflecting better quality [53,54]. Therefore, dietary GP supplementation (10 g/kg) could potentially improve some of the egg quality parameters by positively influencing antioxidant status and intestinal morphology of hens, as already observed in our current study.

In the current study, dietary GP supplementation (10 g/kg) increased the gizzard organ index in hens compared to the CN group. This could be attributed to the bioactive compounds found in ginger, like gingerols, shogaols, and zingerone, which are known to stimulate the secretion of digestive enzymes [24,55,56], potentially increasing the mechanical workload of the gizzard, leading to muscular hypertrophy [23]. Additionally, ginger is rich in fiber; dietary fibers have been shown to increase the weight and grinding efficiency of the gizzard [57]. Therefore, it can be hypothesized that dietary GP promotes gizzard development and enhances the production performance of hens. However, no significant effect of dietary GP supplementation on the heart index of hens was observed, although a dose-dependent increasing trend in heart weight was noted. Similarly, dietary GP supplementation did not significantly alter the absolute or relative weight of the hens’ liver compared to the control diet, which partially conflicts with previous reports by [58], which suggested that dietary phytogenic premixes could improve the relative liver weight of broilers. However, dietary GP does not adversely affect liver health; rather, it preserves the liver’s normal morphology better than the CN diet. Furthermore, it was observed that hens fed dietary GP supplementation (10 g/kg) exhibited healthier liver structures with normal hepatocytes and central veins. These observations could be attributed to ginger’s antioxidant capacity, which reduces inflammation and oxidative stress [24]. This aligns with previous findings by [59], who reported that Tribulus Terrestris powder (0.75 g/kg) claimed to improve the liver morphology of broilers. Accordingly, it is reasonable to expect that, in the long term, dietary GP may enhance liver morphology in hens, as it has already been observed to enhance the activity of antioxidant enzymes and also enhance intestinal morphology in our current study.

In addition to the reproductive and endocrine systems [60], the morphological condition of the intestinal tract, evaluated by VH, CD, and the VH/CD ratio, is vital for the health and production capacity of poultry [61]. The state of the villi influences nutrient uptake, which is essential for production [32], as also demonstrated by our current study’s results. Phytogenic compounds from plant sources, used as alternatives to antibiotics, are suggested to support intestinal health in animals. This may enhance nutrient utilization, boosting productivity and egg quality [18]. Our current findings showed that dietary GP supplementation (5 and 10 g/kg) linearly improved the intestinal morphology of egg-laying hens. Consistent with the current results, dietary GP supplementation has been shown to improve intestinal morphology in broilers [22,35]. Moreover, ref. [18] noted that phytogenic feed additives can increase jejunal VH and reduce CD in Japanese quail breeders. The improved intestinal morphology could be linked to ginger’s antioxidant capacity, which may promote gut health and enhance nutrient digestion and absorption, potentially leading to better growth and development [62]. Therefore, the current results suggest that supplementing the diet with GP (10 g/kg) positively influences the morphology of the duodenum, jejunum, and ileum, thereby enhancing gut health and nutrient absorption efficiency in laying hens, which improves hen productivity.

In biological systems, reactive oxygen species are constantly produced and eliminated; however, certain factors can cause an imbalance between their formation and removal, leading to oxidative stress [2]. The prevention of oxidation and the removal of reactive oxygen species from cells are regulated by antioxidant enzymes. These enzymes are either produced by the body’s cells (endogenous) or obtained from the diet (exogenous), converting superoxide radicals and hydrogen peroxide into water and oxygen as end products, thereby safely removing them from the body [63]. Consequently, the antioxidant capacity of laying hens is crucial for controlling their health, production, and egg quality [20]. The levels of antioxidant enzymes such as SOD, CAT, GSH-Px, and T-AOC, along with MDA levels, serve as important indicators for assessing the antioxidant status of animals [64]. Phytogenic substances positively influence hens’ production performance and product quality by enhancing their endogenous antioxidant systems [5]. Our study revealed that dietary GP supplementation linearly increased the hens’ antioxidant enzyme activities (SOD, CAT, T-OAC, and GSH-Px) while linearly reducing MDA activity in a dose-dependent manner. These results suggest that GP enhances hens’ ability to neutralize harmful free radicals and reduce oxidative stress. These findings align with [20], who reported that dietary ginger extract (GE) supplementation increased the antioxidant status of Hyline Brown laying hens while decreasing MDA activity, indicating that GE supplementation could enhance the antioxidant capacity of hens. Similarly, ref. [12] found that dietary GP supplementation (5–15 g/kg) improved the antioxidant status of Japanese quails. Further research has demonstrated the beneficial effects of phytogenic feed additives in increasing antioxidant capacity in broiler chickens [65], Japanese quails [18], and also in crayfish [66]. Therefore, these results suggest that dietary GP (10 g/kg) may boost the antioxidant status of hens during the pre-peak laying phase, potentially due to phytochemical compounds in ginger that enhance antioxidant enzyme activity and improve hen health and productivity.

## 5. Conclusions

This study found that dietary GP supplementation in a dose-dependent manner (5 and 10 g/kg) consistently increased antioxidant capacity, gizzard development, and the morphology of the liver and intestines in hens during the pre-peak laying stage. As a result, adding GP to hens’ diets during this stage positively influences feed efficiency, production performance, HU, and eggshell thickness and strength. Notably, 10 g/kg of dietary GP was significantly more effective for most of these improvements than the other levels. Therefore, these findings provide important insights into the biochemical and histological mechanisms through which dietary GP supplementation benefits hens’ production performance and some egg quality parameters. Further research on the molecular effects of dietary GP supplementation on the productive and reproductive performance of hens during the pre-peak laying phase is recommended.

## Figures and Tables

**Table 1:** Ingredients and nutrient levels of the basal feed and ginger powder.

Ingredients	Content (%)	Nutrients **	Content
Corn	69.00	Metabolic energy (MJ/kg)	11.00
Soybean meal	21.00	Crude protein (%)	16.10
Limestone	2.20	Calcium (%)	2.60
Dicalcium phosphate	2.80	Total phosphorus (%)	0.42
Sodium chloride	0.30	Available phosphorus (%)	0.22
Premix *	4.70	Methionine (%)	0.45
		Lysine (%)	0.62
Total	100		
Nutrient composition of ginger powder (% DM ^1^)			
Nutrients	Contents (%)		
Dry matter	90.35		
Moisture	9.65		
Crude protein	5.70		
Ether extract	5.42		
Neutral detergent fiber	8.14		
Ash	4.10		
Nitrogen-free extract	66.99		
Organic matter	84.58		
Unknown substances	2.10		

Note: * premix can provide the following nutrients (for each kilogram of diet): vitamin A, 9000 IU; vitamin D_3_, 500 IU; vitamin E, 20 IU; vitamin K_3_, 1.5 mg; vitamin B_1_, 3 mg; vitamin B_6_, 3 mg; niacin, 30 mg; D-calcium pantothenate, 13 mg; D-biotin, 0.09 mg; folic acid, 0.085 mg; copper, 10 mg; iron, 80 mg; manganese, 50 mg; zinc, 80 mg. ** All the nutritional levels are average calculated values; ^1^ DM, dry matter.

**Table 2:** Effects of supplementing diets with GP on egg quality in Xiaoshan hens (Mean ± SEM).

Items	Groups			SEM ^1^	*p*-Values		
	CN	T1	T2		An ^2^	Ln ^3^	Qu ^4^
Egg weight (g)	48.23	51.79	50.83	1.85	0.1731	0.181	0.180
% Shell weight	12.54	12.53	12.56	0.61	0.9989	0.981	0.969
% Albumen weight	56.84	56.66	56.33	1.98	0.9659	0.798	0.968
% Yolk weight	30.70	30.78	31.21	2.21	0.9690	0.819	0.927
Egg length (mm)	54.07	55.34	55.19	1.13	0.4914	0.339	0.485
Egg width (mm)	39.77	40.83	40.63	0.47	0.0911	0.090	0.145
Egg shape index	73.57	73.83	73.73	1.49	0.9844	0.917	0.889
Albumen height (mm)	4.65 ^b^	4.97 ^ab^	5.62 ^a^	0.35	0.0427	0.015	0.593
Yolk color	14.17	14.50	14.50	0.69	0.8597	0.639	0.786
Haugh unit	70.55 ^b^	71.86 ^b^	77.26 ^a^	2.46	0.036	0.015	0.352
Shell thickness (mm)	0.32 ^b^	0.33 ^ab^	0.39 ^a^	0.02	0.0268	0.013	0.275
Shell strength (kgf)	4.19 ^b^	4.56 ^ab^	4.96 ^a^	0.26	0.0295	0.009	0.942

^a–b^ Values in the same row without a common superscript differ significantly (*p* < 0.05). Data are presented as means ± SEM (n = 6). ^1^ SEM, standard error of mean; ^2^ An, ANOVA; ^3^ Ln, linear; ^4^ Qu, quadratic. Abbreviations: CN, control group, basal diet; T1, basal diet supplemented with 5 g/kg GP; T2, basal diet supplemented with 10 g/kg GP.

**Table 3:** Effect of supplementing diet with dietary GP on the absolute and relative organ indices in Xiaoshan laying hens (Mean ± SEM).

Items	Groups			SEM ^1^	*p*-Values		
	CN	T1	T2		An ^2^	Ln ^3^	Qu ^4^
BW (kg) ^5^	2.49	2.48	2.48	0.06	0.9924	0.916	0.952
Heart weight (g)	6.46	7.46	9.04	0.56	0.1635	0.063	0.796
Heart index (g kg^−1^)	2.60	3.02	3.79	0.29	0.2540	0.108	0.772
Liver weight (g)	35.67	36.41	36.93	0.76	0.8132	0.529	0.948
Liver index (g kg^−1^)	14.48	14.72	15.09	0.43	0.8559	0.586	0.948
Gizzard weight (g)	15.98	18.07	19.95	0.79	0.1186	0.042	0.949
Gizzard index (g kg^−1^)	6.34 ^b^	7.31 ^ab^	8.08 ^a^	0.26	0.0140	0.004	0.818

^a–b^ Values in the same row without a common superscript differ significantly (*p* < 0.05). Data are presented as means ± SEM (n = 6). ^1^ SEM, standard error of mean; ^2^ An, ANOVA; ^3^ Ln, linear; ^4^ Qu, quadratic; ^5^ BW, body weight at the end of the experiment. Abbreviations: CN, control group, basal diet; T1, diet supplemented with 5 g/kg GP; T2, diet supplemented with 10 g/kg GP.

**Table 4:** Effects of supplementing the diet with GP on the morphology of the intestines in Xiaoshan chickens (mean ± SEM).

Items		Groups			SEM ^1^	*p*-Values		
		CN	T1	T2		An ^2^	Ln ^3^	Qu ^4^
Duodenum	VH (µm)	1419.73 ^b^	1430.24 ^b^	1452.43 ^a^	5.08	0.001	0.001	0.193
	CD (µm)	248.29 ^b^	242.18 ^ab^	234.91 ^a^	2.27	0.020	0.007	0.848
	VH/CD	5.72 ^b^	5.91 ^b^	6.18 ^a^	0.07	0.005	0.002	0.584
Jejunum	VH (µm)	877.94 ^b^	884.95 ^ab^	899.92 ^a^	3.71	0.014	0.005	0.406
	CD (µm)	191.05 ^a^	186.10 ^b^	178.93 ^b^	1.96	0.007	0.002	0.616
	VH/CD	4.59 ^b^	4.76 ^b^	5.03 ^a^	0.07	0.003	0.001	0.413
Ileum	VH (µm)	858.32 ^c^	867.81 ^b^	894.31 ^a^	5.46	<0.0001	<0.0001	0.009
	CD (µm)	185.28 ^a^	159.95 ^b^	150.18 ^c^	5.30	<0.0001	<0.0001	0.011
	VH/CD	4.63 ^c^	5.43 ^b^	5.96 ^a^	0.19	<0.0001	<0.0001	0.136

^a–c^ Values in the same row without a common superscript differ significantly (*p* < 0.05). Data are presented as means ± SEM (n = 6). ^1^ SEM, standard error of mean; ^2^ An, ANOVA; ^3^ Ln, linear; ^4^ Qu, quadratic. Abbreviations: CD, crypt depth; VH, villus height; VH/CD, villus height to crypt depth ratio; CN, control group, basal diet; T1, diet supplemented with 5 g/kg GP; T2, diet supplemented with 10 g/kg GP.

## Data Availability

Data will be provided upon request from the first author.
